# Pathogenic Dermatophytes Survive in Nail Lesions During Oral Terbinafine Treatment for Tinea Unguium

**DOI:** 10.1007/s11046-017-0118-8

**Published:** 2017-03-09

**Authors:** Tomoyuki Iwanaga, Tsuyoshi Ushigami, Kazushi Anzawa, Takashi Mochizuki

**Affiliations:** 1 Department of Dermatology, Kanazawa Medical University, Uchinada, Ishikawa Japan; 2 R&D Laboratories, POLA PHARMA INC., 560 Kashio-cho, Totsuka-ku, Yokohama, Kanagawa 244-0812 Japan

**Keywords:** Dermatophyte, Quantitative real-time PCR, Viability, Onychomycosis, Tinea unguium, Terbinafine, *Trichophyton*

## Abstract

Tinea unguium caused by dermatophyte species are usually treated with oral antimycotic, terbinafine (TBF). To understand the mechanisms of improvement and recalcitrance of tinea unguium by oral TBF treatment, a method of quantifying dermatophyte viability in the nail was developed, and the viability of dermatophytes was analyzed in toenail lesions of 14 patients with KOH-positive tinea unguium treated with oral TBF 125 mg/day for up to 16 weeks. Mycological tests, including KOH examination and fungal culture, and targeted quantitative real-time PCR for internal transcribed spacer (ITS) region, including rRNA, were demonstrated at the initial visit and after 8 and 16 weeks of treatment. Assays in eight patients showed that average ITS DNA amount significantly decreased, to 44% at 8 weeks and 36% at 16 weeks compared with 100% at initial visit. No significant difference was observed between at 8 and 16 weeks, despite the TBF concentration in the nail supposedly more than 10-fold higher than the minimum fungicidal concentration for dermatophytes. This finding suggests the pathogenic dermatophytes in nail lesions could survive in a dormant form, such as arthroconidia, during oral TBF treatment. Both antimycotic activity and nail growth are important factors in treatment of tinea unguium.

## Introduction

Onychomycosis is the most common nail disorder, with a mean prevalence of 11.4% (95% confidence interval: 9.1–13.6%) calculated from 21 previous reports [1]. The major pathogens of onychomycosis are dermatophyte species, a condition called tinea unguium, which produce arthroconidia from hyphae in some nail lesions [2]. *Trichophyton* (*T.*) *rubrum* and *T. mentagrophytes* are the most frequently isolated dermatophyte species, accounting for about 90% of patients with tinea unguium [3–5].

Most patients with tinea unguium are treated with oral antimycotics. Terbinafine (TBF) is utilized worldwide, and 82% of patients in Japan with tinea unguium treated with oral antimycotics were prescribed TBF [6]. Because the complete cure rate, defined both clinically as 100% cleaning of the toenail and mycologically as negative on KOH examination and fungal culture, reached 55% at week 72 for the patients treated with 250 mg/day TBF for 16 weeks. This rate was significantly higher than that of patients treated with 400 mg/day itraconazole for 1 week every 4 weeks for 16 weeks [7].

The geometric mean minimum fungicidal concentrations (MFC) of TBF have been reported to be 0.004 µg/mL for dermatophyte isolates (*n* = 39) [8], 0.026 µg/mL for *T. rubrum* isolates (*n* = 5) [9], and 0.013 µg/mL for *T. mentagrophytes* isolates (*n* = 18) [10]. Pharmacokinetically, TBF has been detected in toenails after 1 to 2 weeks, with concentrations in the distal nail at week 12 of 0.78 and 0.47 µg/g in patients treated with 125 mg/day and 250 mg/day oral TBF, respectively [11, 12]. Because the concentration of TBF in the nail is 10 times higher than the MFC for dermatophyte species, pathogenic dermatophytes in nail lesions should be completely eradicated by oral TBF treatment. Overall, however, 45% of patients treated with TBF for 16 weeks did not reach achieve complete cure at 72 weeks [7], suggesting that fungal elements survive in nail lesions during treatment. Appropriate methods are therefore needed to evaluate the viability of fungi in nail lesions.

In a preliminary study, we developed a method, using quantitative real-time PCR, to assess dermatophyte viability in vitro [13]. In the present study, we optimized the DNA extraction method to enhance the sensitivity of this assay and evaluated dermatophyte viability in toenail lesions during oral TBF treatment. These findings may enhance understanding of the mechanisms underlying improvement and recalcitrance.

## Materials and Methods

### Fungal Strains

Two clinical isolates of *T. mentagrophytes* var. *interdigitale* (KMU 5224, KMU 6742), identified by colony morphology and PCR–RFLP analysis of internal transcribed spacer (ITS) region in the ribosomal RNA (rRNA) gene [14], were obtained from the culture collection of Kanazawa Medical University. These strains were used for standard curves and to confirm the quantification range of conidia in this study.

### Primer Design

The primers, ITSF and ITSR (Table 1), were designed based on the nucleotide sequence of ITS region in the rRNA gene as previously reported [13]. The primer pair specifically detects *Trichophyton* species and *Microsporum* species; however, it does not produce any amplicon from human DNA. The amplification efficiencies using the template DNA from *T. mentagrophytes* var. *interdigitale* and *T. rubrum* were almost identical.

**Table 1:** Primer sequences

Primer	Sequence
ITSF	5′-AGCCCGGCTTGTGTGATG-3′
ITSR	5′-CATTCGCCTAGGAAGCCG-3′
ITS1	5′-TCCGTAGGTGAACCTGCGG-3′
ITS4	5′-TCCTCCGCTTATTGATATGC-3′

### Standard Curve of Quantitative Real-Time PCR (qPCR)

Template DNA for quantitative analysis was prepared from plasmid DNA, constructed using a TOPO TA Cloning Kit (Invitrogen). The ITS region of *T. mentagrophytes* var. *interdigitale* (KMU 5224) was amplified using the primer pair ITS1 and ITS4 (Table 1) [15] and cloned into the plasmid vector pCR2.1-TOPO. The copy number of template for standard curves was calculated from the size of the cloned plasmid and the DNA concentration. Standard curves were generated using 10-fold serial dilutions, yielding samples containing 10^1^ to 10^7^ copies of ITS DNA per reaction. Assays to generate standard curves were performed using a 7900HT Fast Real-Time PCR System (Applied Biosystems) and SYBR Green PCR Kit (Qiagen) in a total volume of 25 µL containing 5 µL of template DNA, 12.5 µL of SYBR Green Master Mix, and 1 µL of each primer (ITSF and ITSR, 12.5 µM). The amplification protocol consisted of 15 min of denaturation at 95 °C followed by 55 cycles of denaturation for 10 s at 95 °C, annealing for 30 s at 60 °C, and extension for 10 s at 72 °C, with the addition of a dissociation stage for subsequent melting curve analysis. The amplification efficiency of the ITS primer set and the correlation coefficient (R^2^) between copy number of template and cycle threshold (Ct) were determined by linear approximation.

### Nucleic Acid Extraction from Conidia and Quantification

A conidial suspension of *T. mentagrophytes* var. *interdigitale* (KMU 6742) was adjusted to 10^7^ conidia/mL with physiological saline, followed by the preparation of 10-fold serial dilutions, yielding 10^1^ to 10^7^ conidia/mL were prepared. Aliquots of 1 mL of each conidial suspension transferred into 2-mL collection tubes and centrifuged at 18,000 × *g* for 15 min. The supernatant was removed, and 28–32 mg of healthy human clipped nail was added to confirm the effect on fungal DNA extraction and PCR. The tubes containing the conidia were frozen in liquid nitrogen for 15 min, and the conidia were pulverized using a Multi-Beads Shocker with a metal corn (Yasui Kikai, Osaka, Japan) at 1500 rpm for 5 s. DNA was extracted using the DNeasy Plant Mini Kit (Qiagen) according to the manufacturer’s protocol, which included RNase A digestion and elution with 50 µL AE buffer. Aliquots of 5 µL were used as the templates, and qPCR was performed under the same conditions as described above. The ITS DNA copy number of each reaction was calculated from the Ct values and standard curve, and the relationship between ITS DNA and the number of conidia was determined using simple linear regression analysis.

### Viability of Dermatophytes in Clinical Samples

This study enrolled patients with tinea unguium on the big toenail, as confirmed by positivity on KOH examinations, evaluated in the Division of Dermatology, Kanazawa Medical University Hospital, between November 2011 and December 2014. The main exclusion criteria included the use of antimycotic therapy during the previous 12 months, poor nail growth, and nails thicker than 3 mm. After providing written informed consent, patients were treated with oral 125 mg TBF once daily, the approved dose in Japan. Nail specimens were taken from the affected sites at the initial visit and 8 and 16 weeks after initiation of therapy.

Each nail specimen was subjected to KOH examination, fungal culture for dermatophytes with Mycosel agar slants (Becton, Dickinson and Company) and qPCR assays. For qPCR assays, about 10 mg nail was frozen in liquid nitrogen; DNA was extracted as described above, eluted with 50 µL of AE buffer, and used with ITS primer pair ITSF and ITSR in qPCR assays as described above. Copy number per 1 mg nail sample was calculated using standard curves. If sufficient nail specimens could not be obtained for these three tests, qPCR tests were given priority. Significant differences were determined using one-way ANOVA followed by Tukey–Kramer post hoc analysis.

### Ethics

The study was performed according to the guidelines of the Helsinki Declaration and was approved by the Ethics Committee of Kanazawa Medical University.

## Results

### Standard Curve of qPCR (Fig. 1)

A standard curve (*Y* = −3.6813x + 41.614, *R* ^2^ = 0.9999) was obtained in a linear range of 10^1^ to 10^7^ copies for the Ct value versus the copy number of cloned ITS region of *T. mentagrophytes* var. *interdigitale*. The amplification efficiency was calculated as 87% from the standard curve.

**Fig. 1:**
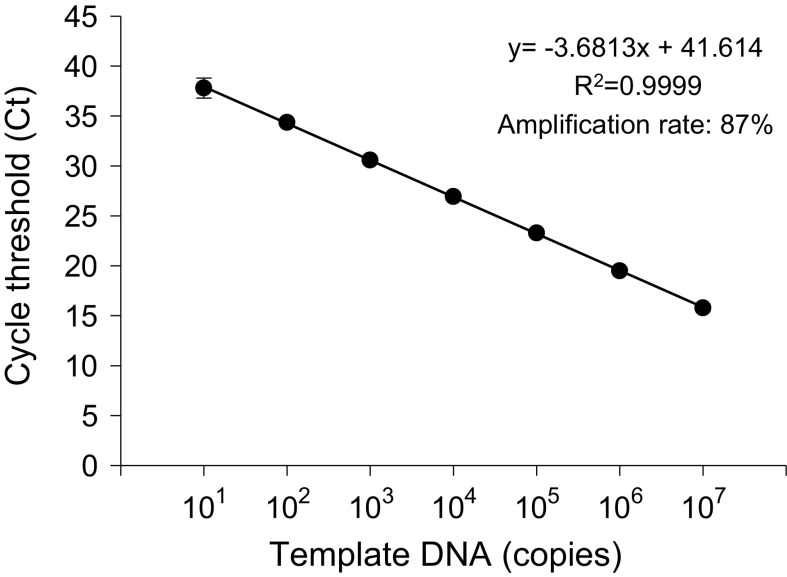
Standard curve for cycle threshold versus template DNA copies by qPCR. Serial dilutions of plasmid DNA containing the cloned ITS region of *T. mentagrophytes* var. *interdigitale* were tested by qPCR using ITS primer pair ITSF and ITSR. Each point represents the mean ± standard deviation (SD) of triplicate measurements

### Nucleic Acid Extraction from Conidia and Quantification (Fig. 2)

The quantification ranges by qPCR also ranged from 10^1^ to 10^7^ conidia when DNA samples were extracted in the presence or absence of human nail. Simple linear regression analysis showed that, under both conditions, the number of ITS DNA copies correlated significantly with the number of conidia (*p* < 0.001 each).

**Fig. 2:**
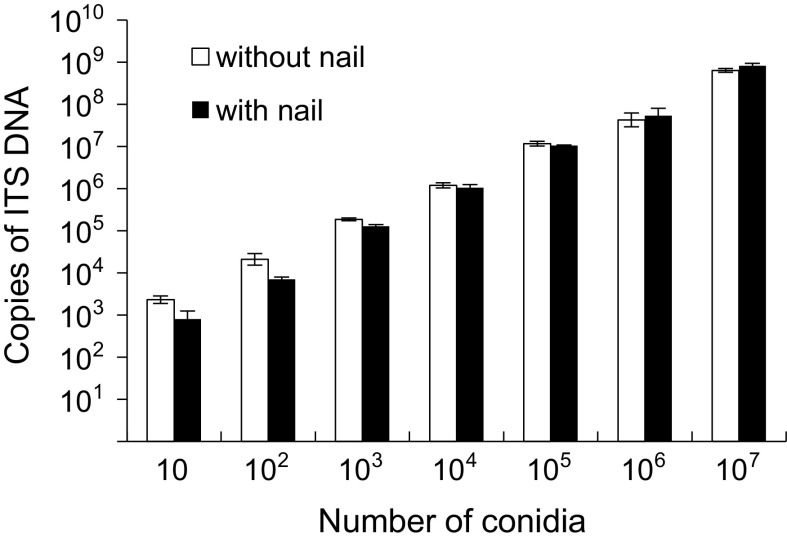
Quantification range of conidia. DNA was extracted from conidia of *Trichophyton mentagrophytes* var. *interdigitale* in the presence or absence of human nail, and ITS DNA copy number was measured by qPCR. Simple linear regression analysis showed that the number of ITS DNA copies correlated significantly with the number of conidia in both the presence and absence of human nail (*p* < 0.001 each). Each bar represents the mean ± standard deviation (SD) of the results of three experiments, with each experiment including duplicate measurements

### Viability of Dermatophytes in Clinical Samples (Table 2)

Fourteen patients with tinea unguium patients (six men, eight women) were enrolled in this study and were treated with 125 mg/day oral TBF, with eight of these patients evaluated after 8 and 16 weeks. The median age of the 14 patients was 72 years (range, 59–83 years) and 13 (93%) also had tinea pedis.

**Table 2:** Demographic and clinical characteristics of the patients included in this study

No.	Age (yr)	Gender	Region	Tinea pedis	Therapy	Assessment		First visit	Second visit 8 weeks	Third visit 16 weeks
1	60	F	R1	+	Terbinafine 125 mg/day	KOH		+	+	N. V.
						Culture		+	N. D.	
						ITS DNA copy number		7811	1788	
2	65	M	L1	+	Terbinafine 125 mg/day	KOH		+	+	+
						Culture		−	−	−
						ITS DNA copy number		18,486	9563	7244
3	76	F	R1	+	Terbinafine 125 mg/day Luliconazole cream	KOH		+	N. V.	N. V.
						Culture		−		
						ITS DNA copy number		2584		
4	64	M	L1	+	Terbinafine 125 mg/day Luliconazole cream	KOH		+	N. V.	N. V.
						Culture		−		
						ITS DNA copy number		9534		
5	75	F	L1	+	Terbinafine 125 mg/day Butenafine cream	KOH		+	+	+
						Culture		−	−	−
						ITS DNA copy number		14,740	9038	6434
6	75	M	L1	+	Terbinafine 125 mg/day Liranaftate cream	KOH		+	N. V.	N. V.
						Culture		−		
						ITS DNA copy number		45,744		
7	59	F	R1	+	Terbinafine 125 mg/day Luliconazole cream	KOH		+	+	+
						Culture		−	−	N. D.
						ITS DNA copy number		14,373	8119	1851
8	66	M	L1	+	Terbinafine 125 mg/day Lanoconazole cream	KOH		+	N. V.	N. V.
						Culture		N. D.		
						ITS DNA copy number		7597		
9	71	M	L1	+	Terbinafine 125 mg/day	KOH		+	+	+
						Culture		−	−	−
						ITS DNA copy number		47,652	25,928	38,434
10	73	F	R1	−	Terbinafine 125 mg/day	KOH		+	−	+
						Culture		−	−	−
						ITS DNA copy number		34,311	18,173	21,561
11	83	F	R1	+	Terbinafine 125 mg/day	KOH		+	+	N. D.
						Culture		−	−	N. D.
						ITS DNA copy number		636,527	155,854	16,097
12	73	M	L1	+	Terbinafine 125 mg/day	KOH		+	N. V.	N. V.
						Culture		+		
						ITS DNA copy number		2446		
13	59	F	L1	+	Terbinafine 125 mg/day	KOH		+	+	+
						Culture		+	−	−
						ITS DNA copy number		66,215	19,559	29,475
14	74	F	L1	+	Terbinafine 125 mg/day Lanoconazole ointment	KOH		+	N. V.	−
						Culture		+		−
						ITS DNA copy number		4819		61
Summary	Average 69.5	Ratio (M:F) 6:8	Ratio (L:R) 9:5	Rate of complications 93%		Positive rate	KOH Culture	100% 31%	88% 0%	86% 0%
	Median 72						DNA	100%	100%	100%

*N*.*D.* not determined; *N.V*. no visit

At the start of the study (initial visit), 14 of 14 (100%) patients were positive on KOH examination, 4 of 13 (31%) were positive for fungal culture, and 14 of 14 (100%) were positive by qPCR. After 8 weeks of treatment, none of the seven evaluated samples was positive for fungal culture, while 7 of 8 (88%) were positive on KOH examination and 8 of 8 (100%) were positive by qPCR. At 16 weeks, none of the six evaluated samples was positive for fungal culture, while 6 of 7 (86%) were positive on KOH examination and 8 of 8 (100%) were positive by qPCR. Although no patient attained a complete cure at week 16, all the observed nails improved from the proximal part.

**Fig. 3:**
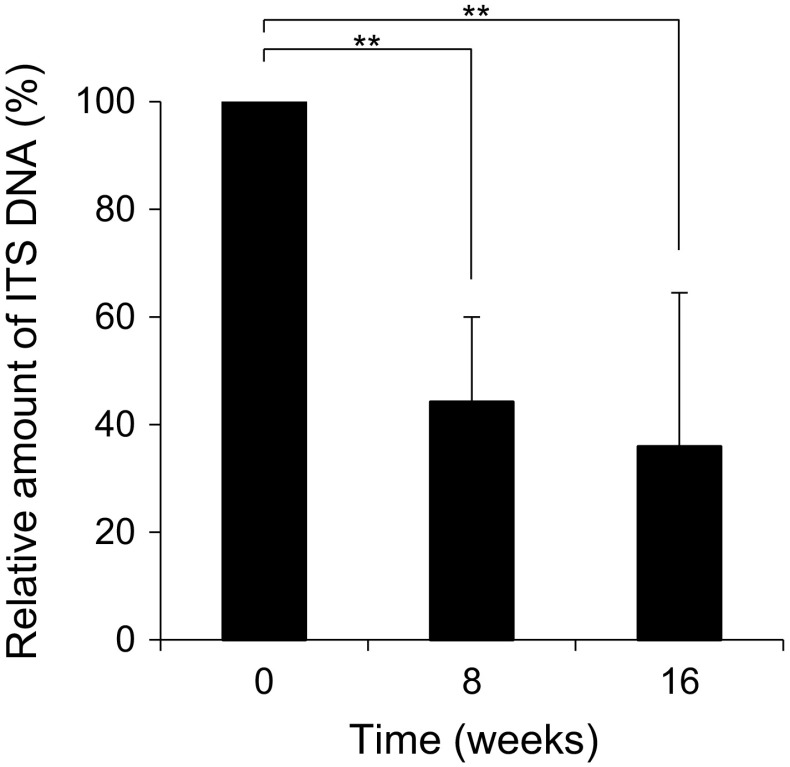
The relative amount of ITS DNA before and after TBF treatment. Patients were treated with oral TBF for 16 weeks. The amount of ITS DNA in nail specimens was measured at 0, 8, and 16 weeks by qPCR and calculated relative to the initial visit (100%). Each bar represents the mean ± standard deviation (SD) of duplicate measurements from eight patients, with results analyzed statistically by one-way ANOVA with Tukey–Kramer multiple comparison tests. **p < *0.01*

At the initial visit, dermatophyte ITS DNA copy number in the 14 nail lesions ranged from 2446 to 636,527 copies/mg. After 8 and 16 weeks of treatment, average ITS DNA amount decreased significantly, to 44 and 36% respectively, compared with 100% at initial visit (*p* < 0.01 by one-way ANOVA with Tukey–Kramer multiple comparison tests). However, no significant difference was observed between at 8 and 16 weeks (Fig. 3).

## Discussion

The ITS primer pair used in this study has a high amplification efficiency, with a detection limit of only 10 conidia following DNA extraction in the presence or absence of clipped nail. In comparison, a nested PCR method had an analytic sensitivity for *T. rubrum* and *T. interdigitale* of 100–1000 cells [16], indicating that our method is more sensitive than other current methods.

Although DNA is more stable than RNA, the number of ITS DNA copies decreased markedly 48 h after adding 1 µg/mL TBF to germinating conidia, with the number of copies consistent with the number of colony-forming units (CFU) [13]. These findings indicate the usefulness of ITS DNA for assessing dermatophyte viability during TBF treatment.

Our results also showed that qPCR was more sensitive than KOH examination and fungal culture [17, 18]. Moreover, the wide range of ITS DNA copy number from the 14 nail lesions at the initial visit was in agreement with previous results in 32 patients with onychomycosis [19].

The average ITS DNA amount at week 16 was 36%, compared with 100% at the initial visit. This finding suggests that about one-third of dermatophytes in the nail specimens survived, despite TBF in the nails possibly being at a concentration 10-fold higher than MFC [8–11]. There are some supportive facts for understanding this result. First, MFC is highly influenced by measurement conditions. The addition of 5% keratin to Sabouraud dextrose broth (SDB) was found to reduce the antimycotic activity of TBF against *T. mentagrophytes* to 1/32 of its initial activity [20]. In addition, we confirmed that the conidia of *T. mentagrophytes* var. *interdigitale* survived more than 2 weeks in saline containing 1 µg/mL TBF, a concentration about 100 times higher than the reported MFC [10], although these conidia were completely eradicated in SDB containing 1 µg/mL TBF (data not shown). Further investigations under conditions similar to the actual nail environment are needed to understand actual MFC.

Second, dermatophytes form arthroconidia from hyphae in some nail lesions [2]. Dormant arthroconidia of *T. mentagrophytes* were found to be resistant to antimycotics, although this resistance was completely lost upon germination [21]. Moreover, the activity of TBF against conidial suspensions of *Microsporum* species was 1/2 to 1/8 than against hyphal suspensions [22]. Complete killing of *T. rubrum* and *T. mentagrophytes* in the dormant phase requires a 1000-fold higher concentration of TBF for than in the growth phase [23]. These reports suggested that the dormant forms such as arthroconidia remain alive during oral TBF treatment in nails which contain abundant keratin.

Consequently, two strategies are suggested to treat tinea unguium, application of antimycotics at a much higher concentration than MFC, and nail growth. Recently, the highly active topical antimycotics efinaconazole and luliconazole, with MFCs against dermatophytes (*T. rubrum* and *T. mentagrophytes*) ranging from 0.0156 to 1.0 and 0.002 to 0.016 µg/mL, respectively, were introduced to treat tinea unguium [24, 25]. Topical treatment with a 10% solution of efinaconazole for 28 days penetrated 6.0 mg/g [26] and could therefore deliver 6000 times higher concentration than MFC to the nail. As other strategy, nail growth promoter may be able to regrow healthy nail during which dermatophyte remains dormant by antimycotics.

In conclusion, dermatophytes in the nail survive during TBF treatment probably caused by producing dormant forms such as arthroconidia. It is suggested that both antimycotic activity and nail growth are important factors in the cure of tinea unguium.
